# Development and Characterization of Scented PLA-Based Biocomposites Reinforced with Spent Coffee Grounds and Lignin for FDM 3D Printing

**DOI:** 10.3390/polym17212836

**Published:** 2025-10-24

**Authors:** Zeineb Siala, Ahmed Koubaa, Sofiane Guessasma, Nicolas Stephant, Ahmed Elloumi, Martin Beauregard

**Affiliations:** 1 Forest Research Institute, UQAT—Université du Québec en Abitibi-Témiscamingue, 445, boul. de l′Université, Rouyn-Noranda, QC J9X 5E4, Canada; zeineb.siala@uqat.ca; 2 Creation and new media, UQAT—Université du Québec en Abitibi-Témiscamingue, 445, boul. de l′Université, Rouyn-Noranda, QC J9X 5E4, Canada; martin.beauregard@uqat.ca; 3 Design, ISAMS—Institut Supérieur des Arts et Métiers de Sfax, 34 Av. 5 août, Sfax 3069, Tunisia; loumiahmed@gmail.com; 4 INRAE, UR 1268 Biopolymères Interactions Assemblages, Équipe Paroi Végétale et Polymères Pariétaux, Site de la Géraudière BP71627, CEDEX, 44316 Nantes, France; 5 Institut des Matériaux de Nantes Jean Rouxel (IMN), Centre National de la Recherche Scientifique (CNRS), Nantes Université, 44300 Nantes, France; nicolas.stephant@univ-nantes.fr; 6 Electromechanical Systems Laboratory LASEM, National Engineering School of Sfax, University of Sfax, Sfax 3038, Tunisia

**Keywords:** scented biocomposites, biodegradable, additive manufacturing, lignin, spent coffee grounds, fragrance additives, fused deposition modeling

## Abstract

This study investigates the development of biodegradable, scented bio-composite filaments incorporating industrial residues, specifically spent coffee grounds (SCG) and lignin (LI), into a PLA matrix for FDM 3D printing. Two fragrance additives, essential oil (EO) and microencapsulated fragrance powder (FP), were introduced (3%) to enhance sensory properties. The research investigates the effects of filler content (5%, 10%, and 15%) and fragrance additives on the surface chemistry (FTIR), thermal stability (TGA and DSC), mechanical properties (Tensile, flexural and impact), microstructure, and dimensional stability (Water absorption test and thickness swelling). Incorporating industrial residues and additives into PLA reduced the thermal stability, the degradation temperature and the glass transition temperature but increased the residual mass and the crystallinity. The effect of lignin was more pronounced than that of SCG, significantly influencing these thermal properties. Increasing the filler content of spent coffee grounds and lignin also led to a progressive decrease in tensile, flexural, and impact strength due to poor interfacial adhesion and increased void formation. However, lignin-based biocomposites exhibited enhanced stiffness at lower concentrations (≤10%), while biocomposites containing 15% SCG doubled their elongation at break compared to pure PLA. Adding fragrance reduced the mechanical strength but improved ductility due to plasticizer-like interactions. Microstructural analysis revealed heterogeneity in the biocomposites’ fracture surface characterized by the presence of pores, filler agglomeration, and delamination, indicating uneven filler dispersion and limited interfacial adhesion, particularly at high filler concentrations. The water absorption and dimensional stability of 3D-printed biocomposites increased progressively with the addition of residues. The presence of essential oil slightly improved water resistance by forming hydrogen bonds that limited moisture absorption. This article adds significant value by extending the potential applications of biocomposites beyond conventional engineering uses, making them particularly suitable for the fashion and design sectors, where multi-sensory and sustainable materials are increasingly sought after.

## 1. Introduction

Additive manufacturing (AD) has revolutionized industries such as aerospace, automotive, fashion, arts, regenerative medicine, and pharmaceuticals. This progress stems from its ability to create complex shapes and materials, enable decentralized production, and support customized manufacturing [1]. Additionally, 3D printing offers significant environmental and economic benefits by reducing material waste, promoting the use of sustainable materials, and optimizing energy consumption [2]. Fused Deposition Modeling (FDM) is the most widely used 3D printing technique due to its ease of use, fast processing, affordability, reliability, and ability to accommodate innovative materials [3]. This method relies on the thermal extrusion of a thermoplastic filament through a nozzle. The filament is heated, melted, and deposited in successive layers to form the desired structure.

Among the most widely used polymers for FDM is polylactic acid (PLA). Derived from renewable resources like corn starch and sugarcane, it offers an environmentally friendly alternative to petroleum-based plastics. PLA stands out as a particularly advantageous material due to its physical properties and biodegradability. It naturally decomposes under appropriate conditions, reducing plastic waste and the ecological footprint of plastic production [4]. Moreover, PLA offers both high mechanical strength and biocompatibility [1]. Its low melting temperature and glass transition temperature make it well-suited for FDM 3D printing [5]. This characteristic promotes smooth and consistent extrusion while ensuring good adhesion to the print bed. Additionally, its inherently flexible nature enhances its compatibility with the printing surface, thereby reducing the risk of deformation. However, PLA is subject to limitations, including its fragility, reduced toughness, limited moisture resistance, and a low crystallization rate [6]. Its low glass transition temperature and poor thermal resistance also restrict its performance under extreme conditions. Additionally, its hydrophilic nature can compromise its stability in humid environments. These limitations hinder its adoption in certain commercial applications that require more resilient and durable materials. The incorporation of natural fibers into PLA offers significant environmental and economic advantages. Indeed, incorporating these fibers can enhance the strength and accelerate the biodegradability of PLA, while maintaining a low cost and lighter weight [7,8]. The development of bio-based materials represents a promising alternative to petroleum-derived plastics, whose widespread use has led to the depletion of landfill capacities and caused significant environmental impacts. For these reasons, several studies have focused on developing PLA-based biocomposites with natural fibers for FDM [9]. This approach highlights the recycling of agricultural [10,11], food [12], and forestry residues [13] through FDM technology, offering promising alternatives for sustainable production practices.

Spent coffee grounds (SCG) are an industrial food waste generated during the preparation of coffee beverages and instant coffee production. The amount of used coffee grounds depends on global coffee consumption, the second most consumed beverage worldwide: approximately 650 kg of used coffee grounds is produced from one ton of green coffee beans [14]. Large quantities are disposed of annually, contributing to environmental issues such as greenhouse gas emissions and depletion of natural resources [15]. The spent coffee grounds possess a lignocellulosic composition, rich in cellulose, hemicelluloses, and lignin [16]. Their high cellulose content contributes to a predominantly crystalline structure, conferring notable tensile strength. Additionally, these residues are known for their water and oil retention capacities, high emulsion activity, and stability [17]. These properties create opportunities for their valorization in the development of biocomposites for FDM. Biocomposites based on coffee grounds and PLA exhibit higher elongation at break compared to pure PLA [12]. Additionally, studies have shown that incorporating coffee grounds into PLA preserves its thermal properties. However, at higher filler contents, a progressive decline in mechanical performance, particularly in tensile strength, is generally observed [18,19]. A recent study on PLA composites containing 10% SCG revealed that the addition of coffee grounds enhances printability by reducing viscosity, improving interlayer adhesion, and minimizing defects [20].

Lignin (LI) is one of the most abundant by-products of wood processing. Approximately 50 million tons of lignin are produced annually, primarily in the form of kraft lignin, organosolv lignin, and lignosulfonate, derived from pulp and paper manufacturing processes. Despite its potential as a renewable biopolymer, lignin remains underutilized, with 98% being burned for energy production and only 2% valorized in other applications [21,22]. Lignin is gaining increasing attention in the fabrication of biocomposites due to its low cost, renewable nature, biodegradability, and high carbon content. It acts as a nucleating agent, promoting composite crystallization and thereby enhancing certain structural properties [23,24]. However, high lignin concentrations can negatively impact mechanical strength and reduce elongation at break, making the bio-composite more brittle and challenging to print using additive manufacturing techniques [25,26].

However, the use of natural fibers remains limited due to the undesirable odors generated during the manufacturing process. These olfactory emissions, primarily caused by the release of volatile organic compounds (VOCs) at high temperatures, can degrade air quality, impact human health safety, and reduce the acceptability of the final products, particularly in enclosed environments such as vehicle interiors [27]. To mitigate these issues, several approaches have been explored, including the use of VOC-absorbing agents to limit malodorous emissions in materials intended for vehicle interior components [28] and the optimization of processing parameters, which established a critical temperature threshold at 210 °C, beyond which odor emissions become problematic [29]. Furthermore, the incorporation of fragrance additives, such as essential oils and microencapsulated perfumes, has been investigated for the development of scented materials [30]. Xiao et al. [31] evaluated the effect of adding microencapsulated fragrance in the production of natural fiber-based wallpaper by analyzing its impact on the material’s chemical and thermal properties. Their results revealed that the chemical structure of the material exhibited no significant interaction with the fragrance additive. Microencapsulation has been shown to enable controlled and prolonged fragrance release, lasting up to three months, which is a significant advantage for applications requiring long-term olfactory stability. In another study, Liu et al. [32] developed scented ABS composites by incorporating essential oils to investigate their impact on the material’s mechanical properties. The findings highlighted a gradual decrease in impact resistance and yield strength with increasing fragrance content, while elongation at break significantly improved. Research on scented composites remains limited and relatively unexplored. Currently, there is no research available on biodegradable scented biocomposites or scented composites specifically designed for FDM 3D printing.

Despite growing interest in biocomposites, studies on sensory-enabled materials remain limited, particularly regarding their integration into additive manufacturing processes. This research addresses this gap by combining eco-friendly materials with olfactory functionality, creating interactive material that engages users beyond visual and tactile dimensions. This study aims to develop a biodegradable and scented bio-composite filament derived from industrial residues, specifically lignin and spent coffee grounds, using PLA as the matrix. Two types of fragrance additives, essential oil and microencapsulated perfume powder (fragrance powder), were incorporated into the bio-composite, with the processing temperature carefully controlled below 195 °C to preserve volatile compounds. The primary objective of this research is to assess the influence of filler type and content, as well as the two fragrance additives, on surface chemistry, thermal properties (DSC and TGA), mechanical performance (tensile, flexural, and impact resistance), microstructure, and water resistance. This research demonstrates the potential of the developed biocomposite for applications in the field of fashion design, where the incorporation of environmentally sustainable and olfactorily active materials offers a novel approach to creating interactive, multi-sensory products.

## 2. Materials and Methods

### 2.1. Materials

The lignin (LI) used is ammonium lignosulfonate, supplied by Borregaard in powder form. Spent coffee grounds (SCG) were collected as post-consumer residues from the Van Houtte café. The chosen matrix for these biocomposites is IngeoTM 3D850 polylactic acid (PLA), produced by NatureWorks (Minnetonka, MN, USA). This grade is particularly suitable for the manufacture of filaments intended for 3D printing by additive manufacturing. It has a fluidity index of between 7 and 9 g/10 min, as well as a density of 1.24 g/cm^3^.

Two types of fragrance additives were used. The first is a fragrance powder (FP) supplied by iSuoChem (Shanghai, China). This powder consists of microencapsulated oil-based fragrance pigments, is environmentally friendly, and exhibits high thermal resistance up to 220 °C. The particle size of the fragrance powder ranges from 5 to 10 μm. The second additive is an essential oil (EO), which is 100% natural and was provided by Botanic Planet (Brampton, ON, Canada).

The preparation of SCG particles was carried out in four steps. First, it was crushed using a domestic mill. A sieve was then carried out using a RO-TAP sieve from W.S. Tyler (Mentor, Virginia, USA), with a 250 μm mesh. Finally, the sieved particles were oven-dried at 60 °C for one week to ensure complete removal of moisture. Lignin was dried for 24 h at 80 °C to a residual humidity of less than 1%. The PLA was dried at 45 °C for 12 h to preserve its viscosity and ensure that the residual humidity is below 0.2%.

The different formulations of the developed biocomposites are presented in Table 1.

Biocomposite production begins with extrusion, which homogenizes components using a Haake PolyLab OS Rheodrive 7 (Waltham, MA, USA), a twin-screw extruder with a temperature profile of 170–185 °C and a rotation speed of 40 rpm. The extrudate was pelletized using a Thermo Scientific standard pelletizer and dried at 60 °C for 24 h with an AIRID dryer equipped with an agitator–rotator system.

Filaments are shaped via a second extrusion using a 3DEVO Composer 450 desktop filament maker (Utrecht, The Netherlands), a single-screw extruder, ensuring homogenization and a consistent diameter of 1.75 ± 0.1 mm. Pellets melt through four heating zones, extrude through a 4 mm nozzle, cool via ventilation, and are stabilized by extraction wheels with real-time optical monitoring. Filaments were wound onto a spool with automatic adjustment. Table 2 lists parameters.

A CreatBot DX Plus printer (Zhengzhou, China) utilizing the Fused Deposition Modeling (FDM) technique was used to fabricate test samples. Sample designs were created in SolidWorks (2018), and printing parameters were configured in Simplify3D (2024). The printer featured a 0.8 mm nozzle, with an extrusion temperature of 200 °C and a bed temperature of 60 °C. Key settings included a 40 mm/s infill speed, 100% infill, 0.4 mm layer thickness, and a ± 45° raster angle.

### 2.2. Biocomposites’ Characterization

#### 2.2.1. Surface Chemistry

Fourier Transform Infrared Spectroscopy (FTIR) analysis was performed with a Shimadzu IR Tracer-100 (Kyoto, Japan) to investigate the surface chemistry of various materials and biocomposites. Before analysis, drying samples at approximately 60 °C in an oven is a necessary step to avoid overestimation of the O–H bonds, observable at 3400 cm^−1^ and 1640 cm^−1^ in the absorption bands. The samples were analyzed using a total attenuated reflectance (TPA) fixture. The wavelengths used range from 4000 cm^−1^ to 400 cm^−1^. Spectra of the different components and biocomposites were obtained with 64 scans at a resolution of 8 cm^−1^.

#### 2.2.2. Thermal Properties

Differential Scanning Calorimetry (DSC) tests were conducted using a TA Instruments Q20 instrument (New Castle, DE, USA) equipped with a cooling accessory for use under a nitrogen atmosphere, according to ASTM D 3417 [33]. Sample masses range from 10 mg to 15 mg. The analysis was conducted under a nitrogen atmosphere using two heating–cooling cycles from 10 °C to 200 °C at a rate of 10 °C/min, each followed by a 3 min isothermal hold to reset the thermal history. This method is suitable for determining the glass transition temperature (T_g), degree of crystallization (X_C), reaction enthalpy, melting temperature (T_m), and crystallization temperature (T_C).

The crystallinity of biocomposites can be calculated according to the following equation:

(1) $$\chi_{cr}(\%)=\;\frac{\Delta H_{f}}{\Delta H_{f}^{0}}\times \frac{1}{\left(1-\omega \right)}\times 100$$

where $\Delta H_{f}$ is the measured melting enthalpy, $\Delta H_{f}^{0}$ is the melting enthalpy of a crystalline PLA (93 J/g), and ω is the mass fraction of fibers in the composite.

The thermal stability of neat PLA, fillers, and filler-PLA biocomposite filaments was evaluated using thermogravimetric analysis (TGA) performed on a Q50 device (TA Instruments). Thermogravimetric analysis (TGA) was conducted in dynamic mode to assess the thermal stability of the samples. The temperature was ramped from 10 °C to 600 °C at a heating rate of 10 °C/min. Sample masses ranged from 10 to 20 mg, ensuring consistency in the analysis. This analysis enables the study of thermal degradation of materials by measuring mass evolution (loss or gain) as a function of time at high sample temperatures under a controlled atmosphere.

#### 2.2.3. Mechanical Properties

The tensile test procedure followed ASTM D638 [34]. guidelines, employing a Zwick/Roell Z020 machine (Ulm, Germany) with a 20 kN load capacity. Strain was monitored using a Zwick/Roell Clip-on extensometer model 5025-1, while data processing was performed using TestXpert software (V12.0). Tensile properties, including Young’s modulus (E), tensile strength at yield, and elongation at break, were measured at a crosshead speed of 2 mm/min and ambient temperature. Testing adhered to ASTM D638 standards [34], with the reported values being the mean of at least five tests.

Three-point flexural testing was carried out as per ASTM D790 to determine the apparent flexural modulus (E), flexural strength, and maximum flexural strain (ε) [35]. The tests were conducted on five samples using the same Universal Testing Machine at a speed of 2 mm/min.

The Izod impact strength of the biocomposites was measured according to the ASTM D256 standard using a Basic Pendulum Impact Tester (BPI-5.5, Zwick Roell Group, Ulm, Germany) equipped with a 2.75 Joule pendulum [36]. The reported impact strength values represent the mean of ten measurements.

#### 2.2.4. Scanning Electron Microscopy (SEM)

This technique was utilized to examine the morphology of filaments and the cross-sections of tensile test specimens using a scanning electron microscope JEOL JSM5800LV (East Lyme, CT, USA). An acceleration voltage of 15 kV was applied to mitigate charging effects, and the specimens were imaged at magnifications ranging from ×18 to ×200. Before analysis, the sample surfaces were coated with a 50 nm carbon layer to ensure conductivity during observation. This step was performed using a Balzers CED 30 carbon evaporator (Balzers, Liechtenstein).

#### 2.2.5. Water Absorption Test (WA) and Thickness Swelling (TS)

The water absorption and thickness swelling of 3D-printed samples were evaluated following the ASTM D570 standard [37]. The samples were submerged in distilled water at approximately 20 °C for a minimum of 60 days. The water absorption percentage and thickness swelling were determined using Equations (2) and (3), respectively. Three replicates were performed for each measurement.

(2) $$WA\;\left(\%\right)=\frac{W_{w}-W_{d}}{W_{d}}\times 100$$

where $W_{d}\;$ is the initial weight of the sample before immersion, and $W_{w}$ is the weight of the sample after immersion.

(3) $$TS\;\left(\%\right)=\frac{T_{w}-T_{d}}{T_{d}}\times 100$$

where $T_{d}$ and $T_{w}$ correspond to the sample thickness before and after immersion, respectively.

## 3. Results

### 3.1. Surface Chemistry

**Figure 1:**
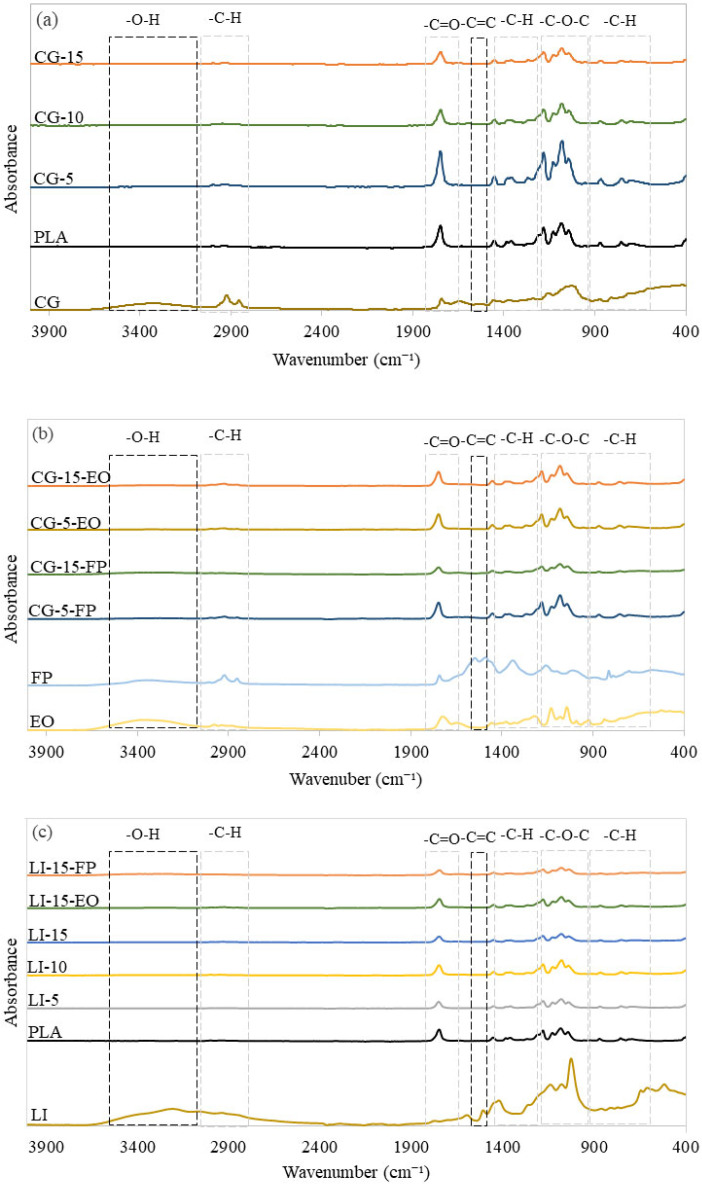
Infrared Absorption Spectra of: (**a**) SCG fillers, PLA and SCG-based biocpmposites, (**b**) perfume additives, PLA, and SCG-EO-based biocomposites and SCG-FP-based biocomposites and (**c**) LI-EO-based biocomposites and LI-FP-based biocomposites.

FTIR analysis evaluates the effect of adding fillers and fragrance additives to the PLA matrix by examining the chemical reactions on the surface of the biocomposites. Figure 1 presents the FTIR spectra of fragrance additives, fillers, pure PLA, and biocomposites within the wavelength range of 400 cm^−1^ to 4000 cm^−1^.

The absorption spectra of PLA exhibit low-intensity absorption bands in the wavenumber range of 3000–3600 cm^−1^, corresponding to the stretching vibrations of hydroxyl (-OH) groups [38]. The bonds within the spectral range of 2863 cm^−1^ to 3000 cm^−1^, were attributed to the stretching vibrations of alkane bonds within the aliphatic (-CH) group, a distinct peak at 1755 cm^−1^ was assigned to the vibration of the carbonyl (C=O) group, which corresponded to the ester bond in PLA monomers [39]. The spectral region between 1456 cm^−1^ and 1278 cm^−1^ was associated with C-H bending vibrations. The absorption bands at 1182 cm^−1^ and 1085 cm^−1^ were attributed to the stretching vibrations of C-O and COC/C-OH bonds, and the peaks at 875 cm^−1^ and 758 cm^−1^ correspond to the amorphous and crystalline phases of PLA, respectively [13].

The FTIR spectra of SCG exhibit a broad absorption band between 3600 cm^−1^ and 3200 cm^−1^, attributed to the stretching vibrations of hydroxyl (O-H) bonds. Two distinct peaks at 2862 cm^−1^ and 2931 cm^−1^ correspond to the stretching vibrations of (-CH_2_) and (-CH) bonds, characteristic of methyl groups from caffeine molecules, sugars, and aliphatic coffee oil [17]. The peak at 1739 cm^−1^ was associated with the symmetric and asymmetric stretching vibrations of carbonyl (C=O) groups, characteristic of hemicellulose and chlorogenic acids, as well as C-N groups from caffeine [40]. The spectral band around 1500 cm^−1^ was attributed to C=C stretching vibrations in the aromatic ring of lignin and the presence of lipids [41]. The absorption band between 950 cm^−1^ and 1219 cm^−1^ corresponded to the stretching vibrations of C-O bonds in the polysaccharide galactomannan within the C-O-H groups. Absorption bands recorded above 900 cm^−1^ were related to the presence of various polysaccharides. Specifically, the peak at 885 cm^−1^ was associated with β-linked D-mannopyranose units, while the peak at 790 cm^−1^ corresponds to α-linked D-galactopyranose units [42].

Lignin FTIR spectrum reveals characteristic absorption bands that reflect its complex chemical structure. The broad bands observed in the 3200–3600 cm^−1^ region was attributed to the stretching vibrations of (O-H) bonds in aromatic and aliphatic structure and the bands located between 2958 cm^−1^ and 2870 cm^−1^ correspond to the stretching vibrations of (C-H) bonds in (CH_2_) and (CH_3_) groups, respectively [43]. The bands at 1782 cm^−1^ and 1612 cm^−1^ are associated with (C=O) bonds [26]. In the 1600–1500 cm^−1^ region, intense bands indicate the deformation vibrations of (C=C) double bonds in aromatic rings [44]. The bands between 1458 cm^−1^ and 1438 cm^−1^ correspond to asymmetric (C–H) vibrations. The peaks at 1276 cm^−1^, 1212 cm^−1^, 1153 cm^−1^, and 1029 cm^−1^ are attributed to the absorption of condensed syringyl and guaiacyl units, the breathing vibration of the guaiacyl ring, the stretching of (C–C) and (C–O) bonds, as well as the in-plane deformation of aromatic (C–H) bonds [45]. Bands recorded above 900 cm^−1^ were attributed to out-of-plane vibrations of aromatic (C-H) bonds, further confirming the presence of benzene rings [46].

The FTIR spectra of the biocomposites exhibit subtle shifts in the PLA band positions as the filler content increases. The hydroxyl (O-H) band of the biocomposites is shifted towards slightly lower wavenumbers and appears broader compared to that of pure PLA. This phenomenon can be attributed to the high reactivity of phenolic hydroxyl groups present in LI and SCG, which interact with the carbonyl groups of PLA to form hydrogen bonds [26,47]. Also, a shift in the carbonyl (C=O) vibration bands was observed, indicating interactions between the carbonyl groups of PLA and the polar compounds present in SCG and LI [45]. When 10% SCG is added, small bands appear in the 1500–1620 cm^−1^ spectral region, while similar bands are observed in the spectra of PLA-LI [23]. These observations suggest that interactions between PLA chains and fillers are limited, as evidenced by the dominant presence of PLA functional groups in the FTIR spectra of the biocomposites.

The spectra of fragrance additives exhibit similar absorption patterns. A broad band located between 3653 cm^−1^ and 3089 cm^−1^ in the EO spectrum, as well as a band between 3579 cm^−1^ and 3132 cm^−1^ in the FP spectrum, is associated with hydroxyl (O-H) bond vibrations. Additionally, the peaks observed at 3010 cm^−1^ in the EO spectrum and 3024 cm^−1^ in the FP spectrum correspond to (=C–H) bond vibrations. Meanwhile, peaks around 2995 cm^−1^ and 2818 cm^−1^ in the EO spectrum, along with those around 2989 cm^−1^ and 2831 cm^−1^ in the PP spectrum, are attributed to CH_2_ and CH_3_ group vibrations, respectively [48]. The peaks observed around 1745 cm^−1^ and 1751 cm^−1^ in the FP and EO spectra, respectively, were attributed to the stretching vibrations of carbonyl (C=O) groups, suggesting the presence of esterified compounds or acids [49]. Absorptions around 1654 cm^−1^ in the EO spectrum and at 1554 cm^−1^ and 1500 cm^−1^ in the FP spectrum are associated with (C=C) stretching vibrations of aromatic rings, such as phenols [50]. The band between 1487 cm^−1^ and 1395 cm^−1^, along with the peak at 1354 cm^−1^ in the EO and FP spectra, respectively, is attributed to (C-C) stretching vibrations of benzene rings [49]. Peaks recorded at 1226 cm^−1^, 1134 cm^−1^, and 1049 cm^−1^ in the EO spectrum, as well as the band ranging between 1207 cm^−1^ and 1041 cm^−1^ in the FP spectrum, correspond to (C-O) bond vibrations, indicating the presence of terpenoid components [51]. Peaks at 937 cm^−1^ and 906 cm^−1^ in the EO and FP spectra, respectively, were related to (O-H) bending vibrations. Absorptions above 850 cm^−1^ for both additives were attributed to (C-H) bending vibrations of benzene rings and alkenes [52].

The addition of fragrance additives to the biocomposites does not result in significant changes. The spectra of biocomposites containing FP and EO display absorption profiles similar to those of biocomposites without additives. However, a slight shift in the bands associated with hydroxyl groups was observed, accompanied by moderate broadening, likely due to interactions [53]. The bands corresponding to (C=C) groups observed in biocomposites containing additives are broader than those in biocomposites without additives [54]. The bands corresponding to (C=C) groups observed in biocomposites containing additives are broader than those in biocomposites without additives [54]. Xiao et al. [31] demonstrated that the addition of fragrance additives during wallpaper manufacturing did not lead to significant changes in the infrared spectra of microencapsulated aromatic wallpaper. Thus, the aromatic treatment relies primarily on physical intermolecular interactions, such as hydrogen bonding or Van der Waals forces. Thus, the main difference between standard wallpaper and microencapsulated aromatic wallpaper lies in the perceived scent rather than any alteration of its chemical structure.

### 3.2. Thermal Properties

**Figure 2:**
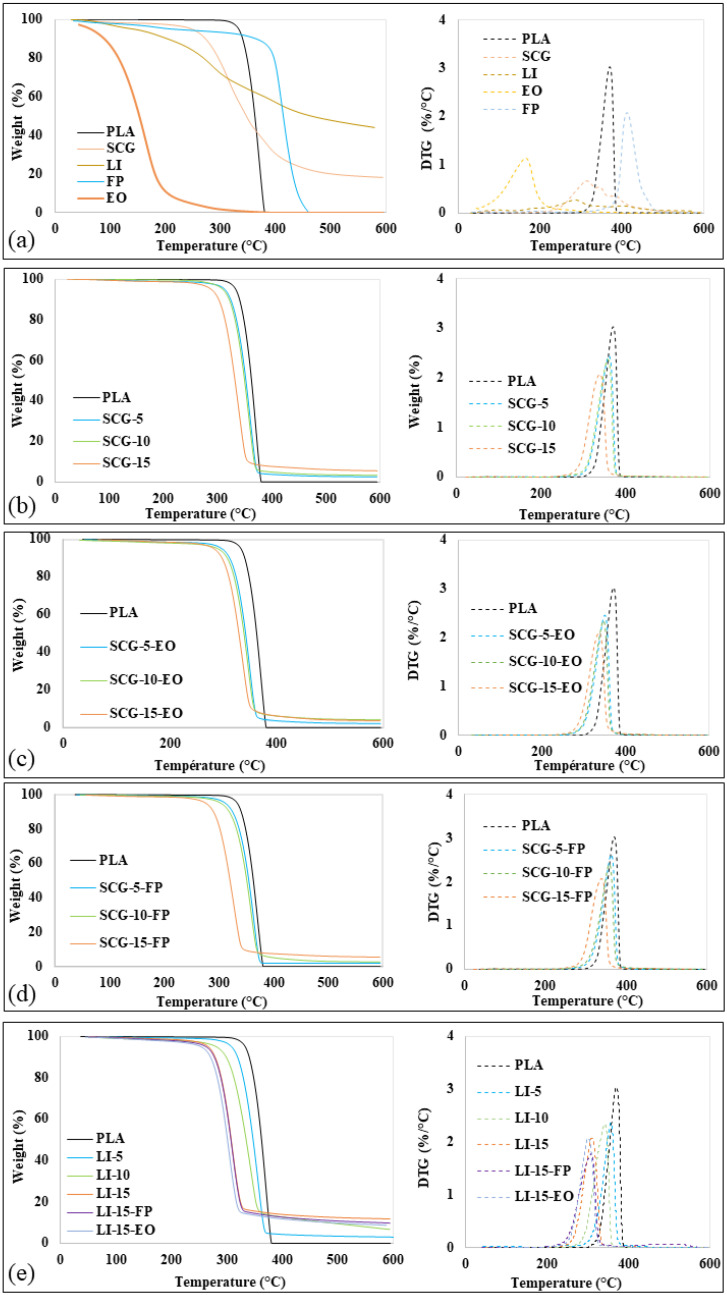
Thermogravimetric analysis: (**a**) ATG and DTG of PLA, fillers and fragrance additives, (**b**) ATG and DTG of PLA and SCG-based biocomposite, (**c**) ATG and DTG of PLA and SCG-EO-based biocomposites filaments, (**d**) ATG and DTG of PLA and SCG-FP-based biocomposites filaments and (**e**) ATG and DTG of PLA and LI-based biocomposites filaments, LI-EO-based biocomposites filaments and LI-FP-based biocomposites filaments.

Figure 2 presents the TG and DTG curves of the fillers, fragrance additives, and bio-composite filaments. The thermogravimetric curves of spent coffee grounds (SCG) and lignin (LI) reveal three distinct phases of mass loss. The onset temperature ($T_{onset}$), the temperatres corresponding to 5% and 20% weight loss ($T_{5\%}$, $T_{20\%}$), the maximum degradation rate temperature $T_{max}$, and the residual weight at 600 °C are presented in Table 3.

The first phase, occurring between 50 °C and 100 °C, corresponds to the evaporation of residual crystalline water [17]. The second thermal phase, which extends from 100 °C to 460 °C, is associated with the degradation of organic constituents. Lignin begins to lose mass at a lower temperature than coffee grounds, indicating that lignin has lower thermal stability compared to coffee grounds. This phase is primarily attributed to the degradation of carbohydrates [23]. Above 340 °C, SCG exhibits a more significant mass loss than lignin, suggesting a higher content of organic matter that decomposes more easily at high temperatures [42]. This degradation results from the progressive decomposition of lignocellulosic components: hemicellulose, which degrades between 200 °C and 340 °C, cellulose, which decomposes between 310 °C and 380 °C, as well as the degradation of certain oils present in SCG [42,55]. The last phase of thermal decomposition begins at 460 °C, with mass losses of approximately 81.58% and 55.12% for coffee grounds and lignin, respectively. This degradation leads to the formation of a condensed aromatic structure, which can generate char [25,47]. In contrast, the thermogravimetric analysis (TGA) curve of pure PLA exhibits a single degradation phase, starting at approximately 330 °C and resulting in complete mass loss, reaching 100% degradation at 400 °C. This behavior is related to the breakdown of the main polymer chain, particularly the ester bond [56].

The thermogravimetric analysis (TGA) curve of essential oil (EO) shows that as the temperature increases, the weight of aromatic substances gradually decreases. The degradation rate of the oil occurs earlier than that of fragrance powder (FP), with the onset of degradation observed in the temperature range of approximately 55 °C to 250 °C. This behavior could be attributed to the loss of adsorbed water and the presence of volatile components in the additives [31]. The TGA curve of fragrance powder (FP) exhibits two distinct decomposition phases. The first phase occurs above 360 °C, leading to a 10% weight loss relative to the initial mass. The second phase takes place between 360 °C and 460 °C, with the maximum thermal decomposition rate observed at 400 °C.

The increase in filler content has a significant impact on the thermal stability of PLA. The increase in filler content has a significant impact on the thermal stability of PLA. The onset degradation temperature, the maximum degradation temperature, and the temperatures corresponding to 5% and 20% mass loss of the biocomposites gradually decrease as the filler content increases. This reduction is attributed to the lower thermal stability of the fillers compared to PLA, as well as to the incompatibility and weak interfacial bonding between the fillers and the polymer [13]. Additionally, the residual mass of the biocomposites increases with higher filler content. This behavior is primarily due to the breakdown of lignin molecular bonds, resulting in the formation of carbonaceous residues and volatile gases during degradation, which increases the proportion of residual material after heating [45]. In the case of coffee grounds (SCG), the residual mass is linked to the presence of extractives, mainly polysaccharides, and the ash content of natural fillers [42]. Incorporating lignin (LI) into PLA results in a more pronounced reduction in the thermal decomposition temperature of the biocomposites compared to SCG. This phenomenon is explained by the earlier thermal degradation of lignin within the polymer matrix, which is associated with active pyrolysis and oxidation processes occurring at lower temperatures than those of PLA [25,26].

The incorporation of fragrance powder (FP) into the biocomposites did not significantly affect their thermal stability, which can be attributed to the higher thermal degradation resistance of FP compared to the other fillers. However, the addition of essential oil led to a slight decrease in the thermal decomposition temperature. This effect is attributed to the presence of plasticizing molecules, which influence the thermal behavior of the polymer matrix [57,58,59].

Table 4 provides a summary of the Differential scanning calorimetry (DSC) analysis of PLA filaments and bio-composite filaments. The first section presents the second heating cycle, including the glass transition temperature ($T_{g})$, cold crystallization temperature ($T_{cc}$), melting temperature ($T_{m}$), cold crystallization enthalpy $\left(\Delta H_{CC}\right)$, melting enthalpy $\left(\Delta H_{m}\right)$, and crystallinity degree $\left(X_{C}\right)$. The second section corresponds to the second cooling cycle, including the crystallization temperature ($T_{c}$), and the crystallization enthalpy $\left(\Delta H_{c}\right)$.

**Figure 3:**
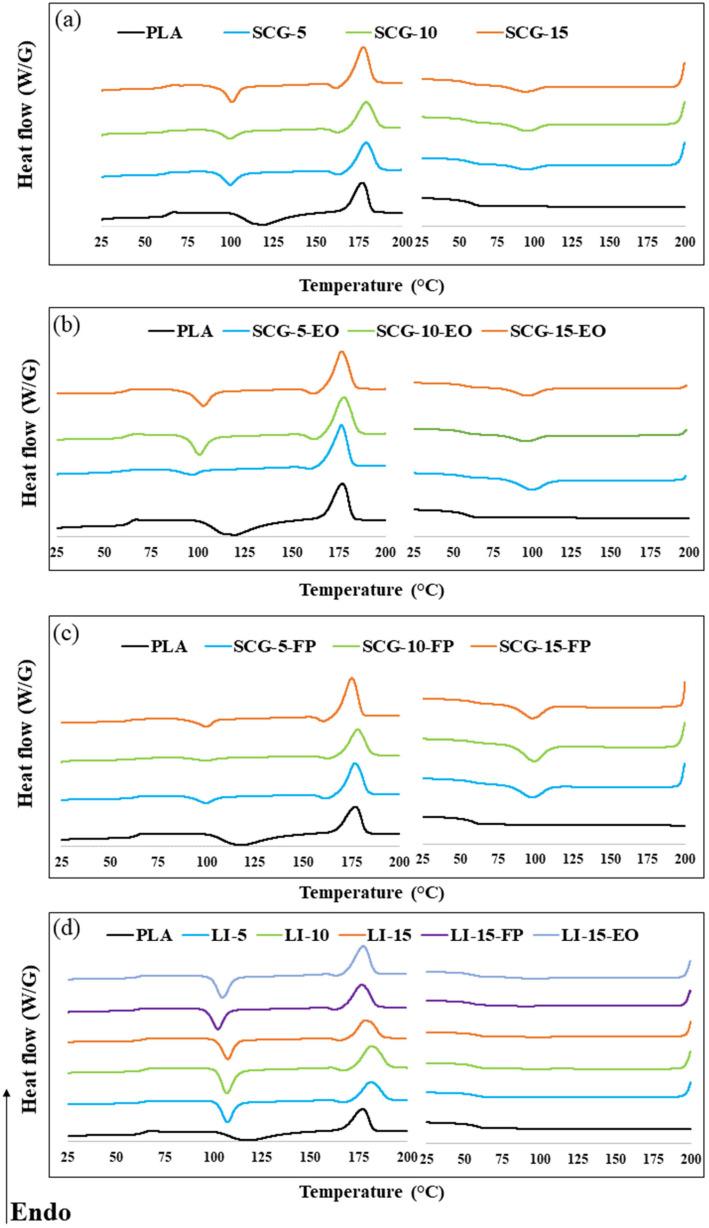
Differential scanning calorimetry of (**a**) PLA and SCG-based biocomposites filaments, (**b**) PLA and SCG-EO-based biocomposites filaments, (**c**) PLA and SCG- FP-based biocomposites filaments and (**d**) PLA and LI-based biocomposites filaments, LI-EO-based biocomposites filaments and LI-FP-based biocomposites filaments.

Figure 3 illustrates that increasing the filler content leads to a slight decrease in the glass transition temperature. Similar findings have been reported in recent studies on lignin- and coffee ground-based biocomposites, confirming that this reduction could be attributed to the poor compatibility between the filler and the matrix or to various molecular factors. These factors include the formation of hydrogen bonds between the carbonyl groups of PLA and the hydroxyl groups of lignin, as well as crosslinking density [23,25].

The crystallization temperature of PLA decreases by approximately 20 °C with the incorporation of fillers. Mimini et al. [24] observed a reduction in T_cc, from 130 °C to 120 °C with the addition of kraft lignin and to 115 °C with the incorporation of lignosulfonate into the PLA matrix. This phenomenon confirms that lignin influences crystal nucleation and growth, potentially promoting recrystallization or the formation of thicker crystalline structures [44,60]. Additionally, studies on PLA–spent coffee ground-based biocomposites have reported similar results, suggesting that this decrease is attributed to the nucleating and plasticizing effects of coffee grounds [18,61,62]. Biocomposites exhibit higher crystallinity levels compared to pure PLA, with this increase being proportional to the filler content. Lignin-based biocomposites demonstrate higher crystallinity than spent coffee ground-based biocomposites. This observation was further confirmed by mechanical resistance tests, which revealed that lignin-based biocomposites possess greater stiffness compared to those containing coffee grounds [63]. The increase in crystallinity could be attributed to the enhanced availability of nucleation sites, thereby promoting faster transcrystallinity and the formation of spherulites [64]. The melting temperature of PLA was not significantly affected by the incorporation of residues and additives, remaining stable at approximately 177 ± 2 °C. During the second cooling cycle, the DSC curve of PLA did not exhibit a crystallization peak, indicating its low crystallization ability [65]. Similarly, lignin-based biocomposites did not show any crystallization. In contrast, crystallization peaks were observed in spent coffee ground-based biocomposites, suggesting an influence of SCG on the nucleation process.

The incorporation of fragrance additives leads to a decrease in the glass transition temperature compared to biocomposites without additives. This effect can be attributed to the plasticizing action of essential oil and fragrance powder [49]. Plasticizing molecules increase the intermolecular spacing between polymer chains, thereby reducing intermolecular forces. As a result, polymer chain mobility is enhanced, facilitating the glass transition process [57]. This process involves a transition from the movement of small mobile molecular units to a segmental motion of the polymer chains, resulting in a decrease in the glass transition temperature [12,19,52]. Additionally, the crystallization temperature slightly increases with the addition of additives. This phenomenon is linked to the presence of oil crystals within the additives, which act as nucleating agents, thereby increasing the degree of crystallinity [66].

### 3.3. Mechanical Properties

Table 5 summarizes the mechanical properties of PLA and its bio-composite formulations reinforced with spent coffee grounds (SCG) and lignin (LI), and fragrance additives. The incorporation of spent coffee ground particles significantly reduces the stiffness of the biocomposite.

Regarding the tensile performance, Young’s modulus of pure PLA decreases by 35% when 15% coffee grounds are added. The inclusion of 10% spent coffee grounds leads to a reduction in tensile strength compared to the 5% spent coffee ground composite. The maximum stress value decreases from 47.57 MPa to 33.62 MPa as the spent coffee ground content reaches 15%. These findings align with previous studies reporting a decline in Young’s modulus and tensile strength due to the difficulty of dispersion, which leads to particle aggregation and poor interfacial adhesion between the matrix and the filler [12,19]. Xiao et al. [47] demonstrated that the ester groups present in SCG oil contribute to particle agglomeration, resulting in a significant reduction in composite strength and stiffness. Similarly, Gama et al. [67] found that SCG oil acts as a plasticizer when incorporated into PLA–cellulose-based composites. It weakens intermolecular polymer bonds, thereby reducing the composite’s rigidity and tensile strength. However, the addition of 15% spent coffee grounds in PLA doubled the elongation at break, increasing from 1.6% to 4.14% compared to pure PLA. These results are consistent with those of Yu et al. [12], who observed a fivefold increase in elongation at break with the incorporation of 15% coffee grounds. This enhancement is attributed to the natural oil content in coffee grounds, which acts as a plasticizer, improving polymer chain mobility and increasing ductility.

Lignin-based biocomposites exhibited a different trend compared to those based on spent coffee grounds. The incorporation of the lignin increased composite stiffness up to 10% lignin content. The Young’s modulus of the 5% lignin biocomposite increased by 9% compared to pure PLA. The enhanced rigidity at low lignin concentrations can be attributed to the presence of phenyl groups in lignin [68] or the constraint effect exerted by lignin within the PLA matrix [25]. However, the maximum stress of PLA decreases after the incorporation of 10% and 15% lignin by weight. Spiridon et al. [69] demonstrated that the addition of 10% lignin reduces tensile strength, dropping from 49.57 MPa to 12.8 MPa. This reduction is attributed to the lack of interfacial bonding between the constituents, particularly due to the hydroxyl groups in lignin macromolecules and the carbonyl groups in PLA [70]. High lignin content creates stress concentration points, leading to lignin agglomeration within the matrix [60]. Furthermore, the incorporation of lignin into PLA results in a reduction in elongation at break. When the lignin content reaches 15%, the elongation at break decreases by 60% compared to pure PLA. Spiridon and Tanase [46] suggested that this phenomenon is due to hydrogen bonding and polar interactions between lignin and the matrix. These interactions restrict the ductile flow of polymer segments, limiting deformation under mechanical stress.

**Figure 4:**
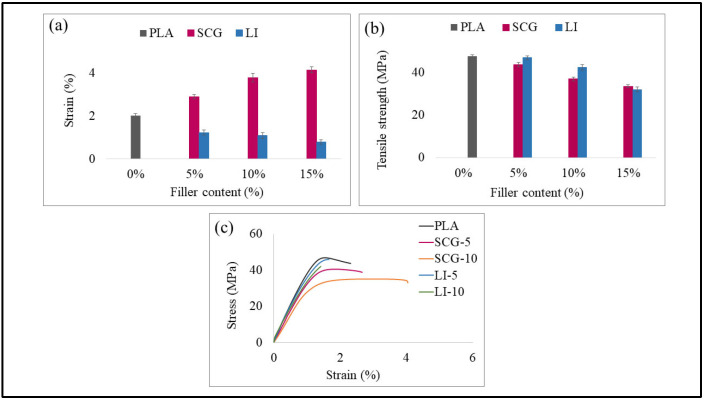
(**a**) Elongation at break of PLA and biocomposites, (**b**) Tensile Strength of PLA and biocomposites, (**c**) Stress–strain curves of PLA and biocomposites.

Lignin-based biocomposites exhibited higher stiffness and tensile strength up to 10 wt% filler content, whereas SCG-based composites showed greater ductility and elongation at break due to the plasticizing effect of natural oils (Figure 4). The standard deviation percentages for the tensile strength and elongation at break of PLA are approximately 3% and 5%, respectively. For the biocomposites, these values reach a maximum of 4.5% for tensile strength and 6.23% for elongation at break. Compared to PLA, these relatively low values indicate good reproducibility of the mechanical measurements and a consistent, stable behavior of the biocomposites.

The incorporation of fragrance additives into spent coffee grounds-based biocomposites with PLA resulted in a further decrease in the rigidity and strength of the composites. The data indicate a moderate reduction in Young’s modulus compared to biocomposites without additives, while tensile strength exhibits a significant decline upon the introduction of these additives. Commercial fragrance powders are typically produced through the encapsulation of essential oil compounds containing aromatic and volatile molecules [71], as evidenced by the FTIR results. Consequently, this reduction in mechanical properties could be attributed to the formation of a “plasticizer-plasticizer” interaction that disrupts the polymer matrix structure [66]. Alternatively, it may be linked to a decrease in molecular weight during microfluidization [72]. Furthermore, the presence of fatty acid ester groups within the essential oils weakens the interfacial adhesion between the filler and the polymer matrix [57]. At a filler content of 15%, Young’s modulus decreased by approximately 33% for lignin-based biocomposites and 41% for spent coffee grounds-based biocomposites. The more pronounced reduction observed in SCG-based biocomposites may be attributed to the excess plasticizer generated by the high content of SCG and fragrance additives. This surplus plasticizer can manifest as a dispersed phase within the PLA matrix, disrupting homogeneity and ultimately reducing tensile strength [73]. Regarding elongation at break, the incorporation of fragrance additives enhanced the ductility of the biocomposites due to the plasticizing effect of the additives, which facilitates polymer chain mobility [49,74]. Elongation at break values exhibited a slight increase in LI-15-FP and LI-15-EO biocomposites compared to LI-15. However, no clear trend was observed as a function of filler content. A peak was recorded at 5% for SCG-FP composites, exceeding 7%. At 10% and 15% filler content, deformation stabilized around 5%. In contrast, SCG-EO biocomposites exhibited a decrease in elongation at 15% filler content. These findings suggest that the saturation of plasticizing molecules, stemming from coffee oil and fragrance additives, may lead to phase separation, negatively impacting ductility [57].

Concerning flexural properties, the stiffness and strength of spent coffee grounds-based biocomposites decrease progressively with increasing SCG. This reduction was attributed to the presence of plasticizing molecules in both the spent coffee grounds and the fragrance additives, which significantly influence the mechanical properties of the biocomposites, as previously mentioned. The Young’s modulus of pure PLA decreased from 3.12 GPa to 1.95 GPa after the incorporation of 15% SCG. When the filler content exceeds 10%, tensile strength undergoes a substantial decline, dropping from 73.75 MPa at 5% SCG to 37.04 MPa. The addition of 10% SCG resulted in a notable increase in ductility compared to pure PLA, with an improvement from 5.16% to 7.47%. This enhancement is attributed to the presence of coffee oil components, which increase the material’s flexibility [56].

For lignin-based biocomposites, the highest Young’s modulus was observed at 5% lignin content, representing a 25% increase compared to pure PLA. However, the incorporation of 15% lignin led to a reduction in elastic modulus. Regarding flexural strength, the maximum stress progressively decreased with increasing lignin content. The flexural strength value declined from 90 MPa to 65 MPa when lignin content was raised to 15%. The study by Kuciel et al. [64] and Kartal and Kaptan [75] confirmed the negative impact of lignocellulosic fiber incorporation on flexural strength. The addition of lignin to PLA resulted in a gradual decrease in elongation at break, indicating a significant reduction in the material’s ductility. The elongation at break values were recorded as 5.16%, 3.20%, 2.33%, and 1.98% for composites containing 0%, 5%, 10%, and 15% lignin, respectively.

The incorporation of fragrance additives exhibited a detrimental effect on stiffness and strength while enhancing the ductility of the biocomposites. SCG-FP and SCG-EO biocomposites containing 5% spent coffee grounds exhibited the highest elongation at break values, reaching 8.28% and 8.91%, respectively. However, at 15% filler content, only a slight increase was observed, similarly to lignin-based biocomposites at the same filler concentration.

As for impact strength, a slight improvement in impact resistance was observed with the incorporation of spent coffee grounds. Specifically, the addition of 5% coffee fibers enhances the impact strength of all three biocomposites (SCG, SCG-FP, and SCG-EO), with values increasing from 15.97% for pure PLA to 16.28% for SCG, 16.04% for SCG-FP, and 15.88% for SCG-EO [74]. This improvement is consistent with the elongation at break results observed in both tensile and flexural tests. However, when the spent coffee grounds content exceeds 10%, the impact resistance of the biocomposites declines compared to pure PLA. This reduction is likely due to limited interactions between the fibers and the polymer matrix, as well as structural defects and void formation within the biocomposite, particularly at high filler concentrations [76]. These structural defects compromise material integrity, thereby reducing mechanical performance [47]. SCG-FP and SCG-EO biocomposites exhibit slightly higher impact resistance than SCG biocomposites. However, the addition of lignin progressively reduces the material’s ability to withstand impacts, with a reduction of approximately 16% and 27% for composites containing 10% and 15% lignin, respectively. These findings align with recent studies [24,77,78], which have confirmed that impact resistance decreases with increasing lignin content. This phenomenon was attributed to poor interfacial adhesion between lignin and PLA, leading to fiber detachment, fracture, and crack propagation [64]. Furthermore, the incorporation of fragrance additives in biocomposites containing 15% lignin slightly increases impact energy compared to composites without additives. Furthermore, the reduction in impact resistance of the biocomposites is associated with the low crystallinity of PLA. This limitation restricts its ability to dissipate impact energy efficiently [79].

The mechanical test results revealed a significant decrease in impact resistance, tensile strength, and flexural strength at high filler concentrations. Several factors contribute to this deterioration. The inherent hydrophobic nature of PLA and the hydrophilic nature of lignin (LI) and coffee grounds (SCG) result in the formation of interfacial voids and poor interfacial compatibility [9]. Additionally, the layer-by-layer deposition process in fused deposition modeling (FDM) often results in imperfect interlayer bonding due to insufficient pressure during extrusion [80]. This mechanism promotes the formation of air pockets and porous structures with large voids [68]. Previous studies have confirmed that printing temperature is a critical parameter in optimizing the mechanical properties of printed composites [81,82]. Yang [83] reported that PLA–hemp composites printed at 230 °C exhibited enhanced interlayer bonding strength compared to those printed at 200 °C. Higher printing temperatures facilitate better fiber/PLA interfacial adhesion, thereby improving bonding between printed layers. Conversely, lower printing temperatures compromise mechanical strength due to increased intra-layer porosity within printed specimens [84]. In this study, the printing temperature was maintained at 200 °C to preserve the volatile compounds in the fragrance additives, which directly influenced the mechanical behavior of the composites. Moreover, the mechanical properties of natural fiber-reinforced filaments are highly dependent on several factors, including fiber length, alignment, and dispersion [13]. During filament fabrication, variations in filament diameter were observed at 15% particle loading, as well as in the presence of fragrance additives. These inconsistencies disrupted filament flow, resulting in inadequate melting between layers and weakened interfacial adhesion.

### 3.4. Biocomposites’ Microstructure

**Figure 5:**
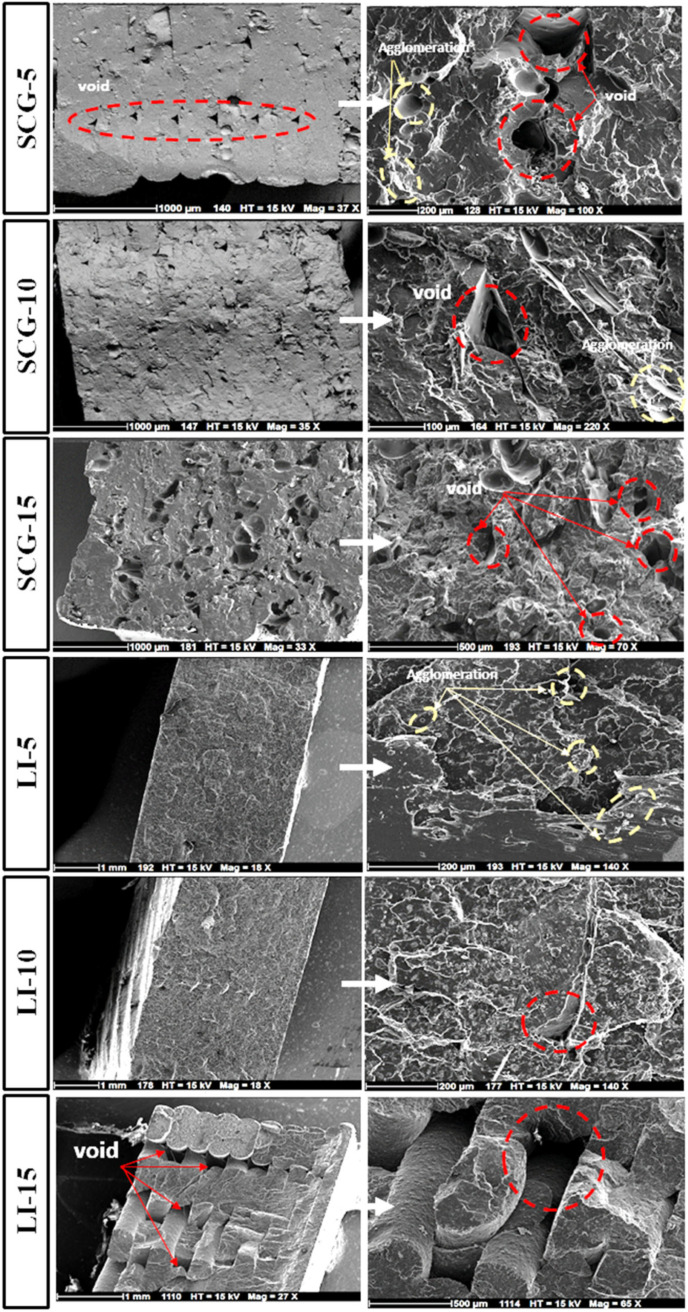
SEM analysis of the tensile fracture surface of the studied biocomposites. The red circles and arrows highlight the voids observed on the fracture surfaces of the biocomposites and the yellow circles indicate the agglomerations observed on the fracture surfaces of the biocomposites.

Scanning electron microscopy (SEM) analysis was conducted on biocomposites incorporating spent coffee grounds, lignin, and fragrance additives. Figure 5 illustrates the effect of increasing filler content on the tensile fracture surface of the printed biocomposites. At filler concentrations below 5%, the number of observed voids remains relatively low, which could be attributed to the discontinuity effect in the extrusion process [85]. This observation aligns with the higher tensile modulus recorded for both types of fillers [62]. However, as the filler content increases, the fracture surface exhibits greater roughness and heterogeneity due to poor adhesion between PLA and the bio-filler, consistent with previous studies on lignin-based composites [40]. This phenomenon can be attributed to the polarity difference between the polymer and the bio-fillers, resulting in phase separation [13]. The presence of micropores in biocomposites with high filler concentrations can be attributed to several factors, including non-uniform particle dispersion during filament fabrication, which contributes to trapped air bubbles, uneven component distribution, and limited connectivity within the matrix [86]. The fracture surface morphology of lignin-based and spent coffee grounds-based biocomposites exhibits distinct differences, likely due to variations in the thermal and dimensional properties of the fillers. These disparities directly influence the mechanical performance variations observed in the results. The SCG-15 biocomposite shows a significantly high porosity [76]. The void size, ranging from 20 µm to 150 µm, may result from particle detachment, where particle dimensions closely match the granularity of coffee grounds. This granularity may contribute to clogging, disrupting material homogeneity, and negatively affecting mechanical properties [20]. Furthermore, the strong particle aggregation observed may be attributed to enhanced interfacial interactions, particularly hydrogen bonding and polysaccharide chain interactions within the matrix [41]. This aggregation contributes to the observed decrease in tensile strength of SCG-based biocomposites [19]. In contrast, lignin-based biocomposites exhibit a more uniform dispersion within the polymer matrix up to a 10% lignin content, suggesting relatively good interfacial adhesion between the polymer matrix and lignin particles. This observation is consistent with the improved Young’s modulus and tensile strength observed for 5% and 10% lignin content. However, the incorporation of 15% lignin revealed interlayer decohesion, leading to the formation of free volume due to the stratification process [68,87]. This behavior may be linked to a reduction in melt flow at the extrusion nozzle, resulting in inadequate adhesion between printed layers [44].

**Figure 6:**
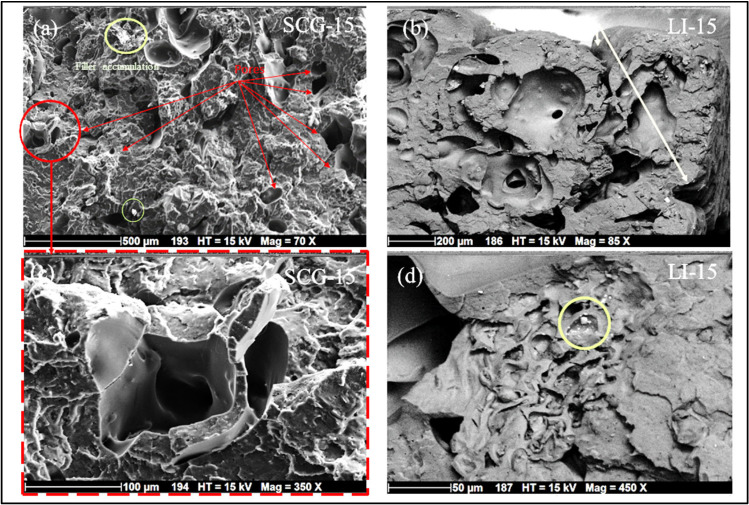
(**a**) SEM image of the fracture surface of the biocomposite containing 15 wt% SCG at 500 µm magnification, SEM image of the fracture surface of the biocomposite containing 15 wt% spent coffee grounds at 500 µm magnification, (**b**) SEM image of the fracture surface of the biocomposite containing 15 wt% LI at 200 µm magnification, white arrows marking regions of weak adhesion between layers, (**c**) presents a magnified view (100 µm) of the red-bordered region in image a, revealing a void surrounded by cracks and (**d**) SEM image of the fracture surface of the biocomposite containing 15 wt% LI at 50 µm magnification. The yellow circles highlight the agglomerations observed on the fracture surfaces of the biocomposites.

Tensile, flexural, and impact strength progressively decrease as the filler content increases. The presence of morphological defects corroborated this reduction in mechanical properties. Figure 5 provides a detailed visualization of these defects in the composite containing 15% fillers. In Figure 6a, red arrows indicate the presence of pores, while Figure 6c presents a magnified view (100 µm) of the red-bordered region in Figure 6a, revealing a void surrounded by cracks. Additionally, green circles highlight the agglomeration of coffee ground particles, suggesting poor dispersion of the filler within the polymer matrix. Furthermore, Figure 6b illustrates interlayer delamination, with yellow arrows marking regions of weak adhesion between layers. These structural irregularities significantly contribute to the deterioration of the biocomposite’s mechanical performance.

**Figure 7:**
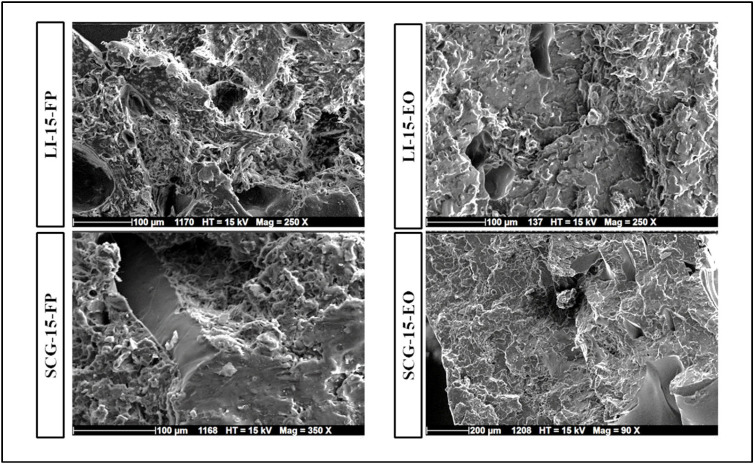
SEM micrographs of the fracture surface of 3D-printed biocomposites with 15% SCG and 15% LI with fragrance additives.

The incorporation of fragrance additives significantly increased the surface roughness of biocomposites (Figure 7). Previous studies have suggested that this trend can be attributed to intensified aggregation and lipid creaming during processing, resulting in surface irregularities [66]. Additionally, the addition of fragrance additives led to an increase in void content. On the one hand, this increase can be attributed to plasticizer saturation, which induces phase separation within the polymer matrix [74,88]. According to Ferri et al. [89], this phenomenon enhances ductility due to its positive effect on polymer chain mobility, reducing polymer–polymer interactions. On the other hand, the rise in porosity attributed to these additives may result from the high concentration of volatile compounds, which evaporate during the extrusion process, leading to pore formation and subsequently altering the material’s internal structure. These microstructural changes highlight the need to optimize processing conditions and filler–matrix interactions to enhance the mechanical performance of biocomposites.

### 3.5. Water Absorption and Thickness Swelling

**Figure 8:**
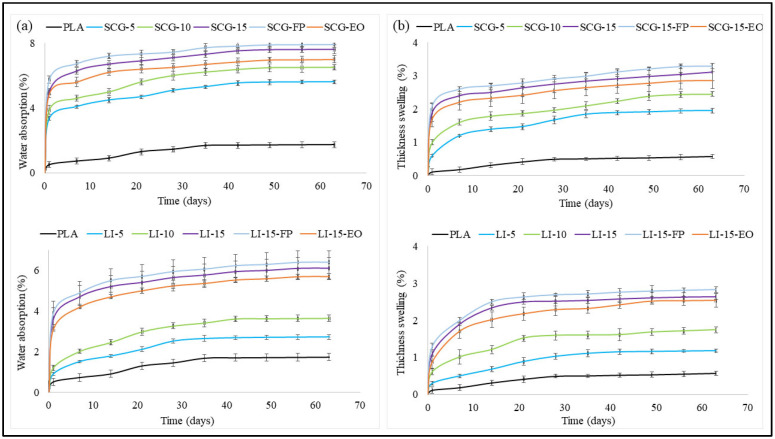
(**a**) Water absorption and (**b**) Thickness swelling of 3D-printed samples.

Thickness swelling and water absorption tests were conducted to evaluate the effect of water molecules on the dimensional stability of the biocomposites. Figure 8a,b depict the variations in water absorption and thickness swelling of PLA and 3D-printed biocomposites after 60 days of water immersion. The graphs exhibited three distinct phases: an initial rapid increase in water absorption and thickness swelling within the first 24 h, followed by a gradual rise over the next seven days. In the final phase, absorption stabilized, reaching saturation, with notable differences observed among the various formulations.

The water resistance of biocomposites was influenced by their morphology, as well as the nature of the polymer matrix and the fillers used. Water absorption tests demonstrated that pure PLA exhibits the highest dimensional stability compared to biocomposites. This behavior was attributed to the hydrophilic nature of the fillers, which promotes progressive degradation under the influence of moisture and temperature [90]. Hygroscopic substances, such as carbohydrates and lignin, increase the composites’ water absorption capacity due to the presence of hydroxyl groups, which form hydrogen bonds with water molecules, as confirmed by results of FTIR analysis [64,91]. Additionally, the fused deposition modeling (FDM) process inherently generates voids and micropores during printing, which contribute to increased water uptake [92,93]. Furthermore, insufficient adhesion between the filler and the matrix facilitates pore formation, enhancing moisture penetration [94]. 3D-printed PLA-SCG biocomposites exhibited higher water absorption and swelling in thickness compared to lignin-based biocomposites (LI). At 10% filler content, water absorption reached 6.51% for SCG-based biocomposites, whereas lignin-based composites showed a lower absorption rate of 3.64%. This difference can be explained by the chemical composition of coffee grounds, which contain fatty acids and caffeine. Caffeine, being a polar molecule with two oxygen atoms, promotes hydrogen bonding with water molecules, thereby intensifying moisture absorption [95]. Moreover, SEM images revealed that at 10% filler content, voids were more prevalent in SCG-based biocomposites compared to LI-based composites. However, at 15% filler content, the water absorption of LI-based biocomposites was slightly lower than that of SCG-based biocomposites. This reduction can be attributed to poor interlayer adhesion, which led to the formation of pores, further promoting water accumulation.

The incorporation of essential oil slightly enhanced the water resistance of the biocomposites. This improvement can be explained by several factors. Essential oil molecules, dispersed at the molecular scale in the PLA matrix, may occupy the interstitial spaces between polymer chains, thereby hindering water penetration [96,97]. Certain molecules in the essential oil, notably aromatic compounds and esters, can form hydrogen bonds with the hydroxyl of fillers (lignin, coffee grounds), thereby enhancing the internal cohesion and limiting water access to hydrophilic sites. In addition, the essential oil contains hydrophobic components, such as terpenes and certain non-polar alcohols, which can create a physico-chemical barrier to water diffusion [98,99]. The intercalation of hydrophobic molecules within the polymer matrix generates localized nonpolar domains that hinder water diffusion, thereby reducing the composite’s overall moisture absorption.

Adding fragrance powder slightly increased water absorption capacity, which could be attributed to the hydrophilic nature of the microcapsules [98]. This effect may also be attributed to the agglomeration of powder particles within the composite matrix, potentially altering the internal structure and facilitating moisture retention.

## 4. Practical Implications

**Figure 9:**
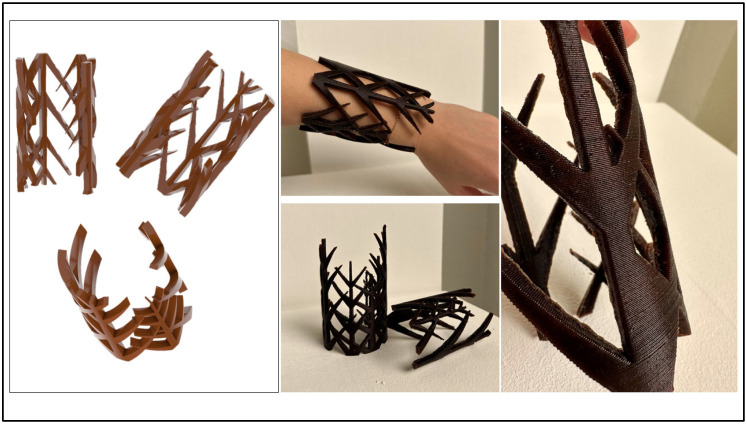
3D-printed fashion accessory produced with the MC-10-EO biocomposite, demonstrating the material’s potential for creative, eco-responsible design applications.

The development of PLA-based biocomposites reinforced with residues offers significant potential for integration within the fields of product and fashion design. By valorizing organic residues through additive manufacturing, this material represents an environmentally responsible alternative to conventional plastics, aligning with contemporary principles of circular economy and sustainable production. In this context, 3D printing emerges as a particularly relevant creative medium, enabling the personalized fabrication of objects while minimizing material waste. The geometric freedom afforded by additive manufacturing expands the designer’s possibilities, fostering the creation of complex, customized accessories that combine aesthetic innovation and ecological awareness. Furthermore, the formulation of scented biocomposites adds an additional sensory dimension to the material. The subtle olfactory qualities derived from the incorporated essential oils contribute to a multisensory experience, enhancing emotional engagement and user interaction with the designed product. This approach positions the biocomposite not only as a structural and sustainable material, but also as a vehicle for sensory and cultural expression, capable of raising environmental consciousness among consumers through both material and experiential qualities. As a practical demonstration, an accessory was fabricated using the MC-10-EO biocomposite (Figure 9).

## 5. Conclusions

This study demonstrated the feasibility of developing biodegradable PLA-based biocomposites reinforced with lignocellulosic residues (spent coffee grounds and lignin) and fragrance agents for FDM 3D printing applications. Spent coffee grounds act as a natural plasticizer, significantly enhancing ductility, particularly at a 15 wt% loading. Conversely, the lignin improved the material’s rigidity at low concentrations (≤10 wt%) but reduced its deformability. From a thermal perspective, the incorporation of these fillers led to a decrease in the thermal stability of PLA, with a more pronounced effect observed for lignin. Crystallinity behavior was improved due to the nucleating action of the fillers, especially lignin, which may partially compensate for the loss in mechanical strength. The addition of fragrance agents, specifically essential oils (EO) and microencapsulated fragrance powders (FP), also impacted the performance of the biocomposites. Both types of additives induced a reduction in mechanical strength, attributed to plasticizing effects that disrupt polymer chain interactions within the matrix. However, they simultaneously enhanced ductility. Water absorption and swelling analysis indicated increased moisture sensitivity with filler addition, particularly in composites containing coffee grounds. Essential oils exhibited a mild hydrophobic effect, thereby limiting water uptake, whereas fragrance powders tended to increase moisture absorption due to their particulate structure. Overall, the combination of bio-based residues and fragrance additives within a PLA matrix presents a promising strategy for tailoring the functional properties of filaments to meet diverse performance requirements. Optimizing formulations and processing conditions remains essential to achieving a balance between mechanical integrity, thermal stability, sensory performance, and environmental durability. Future research should investigate the use of natural compatibilizers or advanced encapsulation techniques to enhance the system homogeneity and improve the overall performance of 3D-printed biocomposites. This research develops a material that is both ecological and sensory, specifically tailored for 3D printing applications. The integration of biodegradable materials with olfactory functionality provides added value, particularly for applications in fashion design and interactive product development. The combination of biodegradability, 3D printability, and sensory functionality offers significant potential for creating innovative and environmentally responsible design solutions.

## Figures and Tables

**Table 1:** Formulations of PLA-Based Biocomposites and Fillers.

Designation	Weight Content (wt%)				
	PLA	SCG	LI	FP	EO
PLA	100	-	-	-	-
SCG-5	95	5	-	-	-
SCG-10	90	10	-	-	-
SCG-15	85	15	-	-	-
SCG-5-FP	95	5	-	3	-
SCG-10-FP	90	10	-	3	-
SCG-15-FP	85	15	-	3	-
SCG-5-EO	95	5	-	-	3
SCG-10-EO	90	10	-	-	3
SCG-15-EO	85	15	-	-	3
LI-5	95	-	5	-	-
LI-10	90	-	10	-	-
LI-15	85	-	15	-	-
LI-15-FP	85	-	15	3	-
LI-15-EO	85	-	15	-	3

**Table 2:** The extrusion parameters of the filament.

Extrusion Parameters	
Extrusion speed	3.5–3.9 RPM
Fan speed	30%
Filament diameter	1.75 mm
Feed zone temperature	170 °C
Melting zone temperature	180 °C
Mixing zone temperature	175 °C
Die temperature	195 °C

**Table 3:** Thermogravimetric analysis measurements of filament specimens.

Formulation	$T_{onset}\;({}^{\circ}C)$	$T_{5\%}\;({}^{\circ}C)$	$T_{20\%}\;({}^{\circ}C)$	$T_{max}\;({}^{\circ}C)$	Carbon Residue (%)
SCG	274.54	248.56	296.66	314.01	18.30
LI	212.99	142.52	269.67	245.57	44.15
FP	394.46	212.37	397.42	314.24	0.02
EO	125.66	65.04	114.09	190.31	0
PLA	342.88	332.58	346.63	370.88	00
SCG-5	333.07	313.08	334.89	360.79	2.5
SCG-10	329.41	309.53	332.25	358.40	3.3
SCG-15	309.13	288.16	313.72	339.36	5.49
SCG-5-FP	336.42	311.92	336.72	363.62	1.9
SCG-10-FP	330.95	303.74	331.86	358.94	2.6
SCG-15-FP	304.04	276.08	301.63	324.35	4.08
SCG-5-EO	323.13	297.83	324.29	350.10	2.1
SCG-10-EO	319.30	290.48	320.45	346.75	3.91
SCG-15-EO	306.86	283.18	310.66	336.63	3.68
LI-5	328.31	301.58	332.81	364.31	4.32
LI-10	312.09	293.91	314.89	341.93	6.76
LI-15	285.10	267.18	290.23	310.12	11.76
LI-15-FP	283.05	263.13	286.94	289.94	10.03
LI-15-EO	279.98	258.64	282.41	282.12	8.54

**Table 4:** Differential scanning calorimetry values of filament samples.

Formulation	$T_{g}\;({}^{\circ}C)$	$T_{cc}\;({}^{\circ}C)$	${\Delta H}_{CC}$ (J/g)	$T_{m}\;({}^{\circ}C)$	${\Delta H}_{m}$ (J/g)	$X_{C}\;(\%)$	$T_{c}\;({}^{\circ}C)$	${\Delta H}_{C}$
PLA	64.3	119.97	34.77	177.02	3.28	33.63	-	-
SCG-5	63.71	99.93	15.2	179.18	31.75	35.93	93.27	5.02
SCG-10	63.5	99.89	11.12	179.04	30.37	36.28	94.19	8.13
SCG-15	62.17	101.07	18.02	178.58	30.03	37.98	94.74	7.96
SCG-5-FP	62.49	100.07	10.29	176.96	32.8	37.12	97.06	14.3
SCG-10-FP	62.6	100.61	4.76	178.49	30.69	36.66	98.61	17.79
SCG-15-FP	61.58	99.92	10.25	175.22	33.67	42.59	97.63	14.36
SCG-5-EO	61.42	99.63	5.85	176.45	33.82	38.27	99.11	12.11
SCG-10-EO	61.71	100.79	18.75	177.93	34.15	40.80	95.24	7.84
SCG-15-EO	61.08	101.01	17.62	176.61	34.38	43.49	96.1	9.19
LI-5	63.43	107.3	25.27	180.46	29.91	33.85	-	-
LI-10	63.85	106.89	28.93	181.74	29.48	35.22	-	-
LI-15	62.99	107.36	23.14	178.73	27.77	35.12	-	-
LI-15-FP	62.48	102.02	20.41	177.14	29.01	36.69	-	-
LI-15-EO	62.12	105.14	20.15	177.75	32.14	40.65	-	-

**Table 5:** The mechanical properties of 3D-printed PLA, biocomposites and biocomposites with fragrance additives as a function of filler content.

	Tensile			Flexural			Impact
Formulation	Tensile Modulus (GPa)	Tensile Strength (MPa)	Strain (%)	Flexural Modulus (GPa)	Flexural Strength (MPa)	Elongation at Break (%)	Impact Strength (KJ/m^2^)
PLA	4.73	47.57	2.01	3.12	90.34	5.16	15.47
SCG-5	3.77	43.79	2.88	2.84	73.75	5.39	16.28
SCG-10	3.32	37.03	3.78	2.33	48.07	6.55	12.17
SCG-15	3.09	33.62	4.14	1.95	37.04	5.32	10.01
SCG-5-FP	3.26	25.44	7.02	2.66	48.58	8.28	16.04
SCG-10-FP	3.07	21.95	5.32	2.08	42.05	6.56	12.29
SCG-15-FP	2.68	19.81	5.06	1.81	36.34	5.49	9.55
SCG-5-EO	3.5	26.01	5.13	2.35	50.19	8.91	15.88
SCG-10-EO	3.21	22.68	4.99	1.89	45.12	7.44	10.95
SCG-15-EO	3.06	20.89	4.2	1.74	31.44	5.78	9.61
LI-5	5.19	46.03	1.23	3.89	81.89	3.84	13.51
LI-10	5.04	42.49	1.09	3.59	78.59	3.33	12.43
LI-15	4.11	31.08	0.78	2.98	65.98	2.91	10.38
LI-15-FP	3.25	22.28	0.88	2.62	51.42	3.06	7.03
LI-15-EO	3.72	21.73	0.81	2.28	55.58	3.12	6.74

## Data Availability

The original contributions presented in this study are included in the article. Further inquiries can be directed to the corresponding authors.

## Abbreviations

The following abbreviations are used in this manuscript:

BC	Biocomposite
EO	Essential oils
FDM	Fused Deposition Modeling
FP	Fragrance powders
LI	Lignin
PLA	Polylactic acid
SCG	Spent coffee grounds
